# Exploring the pathophysiology of post-sepsis syndrome to identify therapeutic opportunities

**DOI:** 10.1016/j.ebiom.2020.103044

**Published:** 2020-10-08

**Authors:** Elisabeth C. van der Slikke, Andy Y. An, Robert E.W. Hancock, Hjalmar R. Bouma

**Affiliations:** a Department of Clinical Pharmacy and Pharmacology, University Medical Center Groningen, University of Groningen, , P.O. Box 30.001, EB70, 9700 RB, Groningen, The Netherlands; b Centre for Microbial Diseases and Immunity Research, University of British Columbia, Vancouver, BC, Canada; c Department of Internal Medicine, University Medical Center Groningen, University of Groningen, Groningen, The Netherlands

**Keywords:** Post-sepsis syndrome, Sepsis, Rehospitalization, Quality of life

## Abstract

Sepsis is a major health problem worldwide. As the number of sepsis cases increases, so does the number of sepsis survivors who suffer from “post-sepsis syndrome” after hospital discharge. This syndrome involves deficits in multiple systems, including the immune, cognitive, psychiatric, cardiovascular, and renal systems. Combined, these detrimental consequences lead to rehospitalizations, poorer quality of life, and increased mortality. Understanding the pathophysiology of these issues is crucial to develop new therapeutic opportunities to improve survival rate and quality of life of sepsis survivors. Such novel strategies include modulating the immune system and addressing mitochondrial dysfunction. A sepsis follow-up clinic may be useful to identify long-term health issues associated with post-sepsis syndrome and evaluate existing and novel strategies to improve the lives of sepsis survivors.

## 1 Introduction

Sepsis is a dysregulated host response to infection that can eventually lead to multi-organ failure (MOF) and is one of the most common causes of death among hospitalized patients [1,2]. Sepsis caused one in five of all global deaths in 2017 (∼11 million deaths/48•9 million cases) [2] and is the most common complication amongst COVID-19 patients [3]. Despite much research, little is known about the precise pathogenesis of sepsis, and therapy remains limited to source control (*e.g.* drainage, antibiotics) and supportive care, [1,4] which can improve mortality and prevent MOF in some, but not all patients, particularly if not administered in the critical early hours [4,5]. There is scarce data that describes the long-term consequences of sepsis and how to optimize health post-sepsis. Mortality rates after surviving the initial sepsis episode remain high: depending on sepsis severity, the one-year post-discharge mortality rate varies between 7-43%, [6] and five-year mortality rate after severe sepsis is 82% [7]. Half of the deaths after sepsis are caused by recurrent infection and cardiovascular events [8]. Long-term mortality is often due to the so-called “post-sepsis syndrome”: a phenomenon defined as consistent physical, medical, cognitive, and psychological issues after sepsis [9]. Post-sepsis syndrome increases readmission risk for infections and the incidence of cognitive impairment, mental health problems, renal failure, and cardiovascular events, compared to non-sepsis hospitalized patients [[10], [11], [12], [13]]. Here, we provide a critical summary of the current understanding of the post-sepsis syndrome and discuss opportunities to optimize health and life span after sepsis.

## 2 Rehospitalization risk

Almost a third of all sepsis survivors are readmitted to the hospital within 90 days, [12] while nearly half of the patients over 50 years of age are readmitted within 90 days [11]. Up to a third of these readmissions are due to recurrent sepsis, [11,12] while other common causes are heart failure, pneumonia and acute renal failure (together ∼15%) [11]. Sepsis survivors have a two-fold higher incidence of sepsis and nearly three-fold higher incidence of acute renal failure as compared to age and comorbidity-matched subjects surviving hospitalizations for other acute medical diagnoses [11]. Recurrent sepsis remains a problem years after discharge as over an eight-year period, more sepsis survivors develop recurrent sepsis compared to randomly sampled patients from a health registry (35% versus 4%), [14] while recurrent sepsis caused nearly one third of deaths in sepsis survivors during this period [14]. Thus, rehospitalization and mortality due to sepsis recurrence and non-septic causes constitute a lethal problem for sepsis survivors.

Preventing sepsis recurrence is difficult since the factors that put patients at risk for sepsis are largely the same risk factors for recurrence, such as increased age, cardiovascular and kidney disease, frailty, and cognitive impairment [15]. Moreover, sepsis induces a state of persistent low-grade inflammation, [16] prolonged immune dysregulation, [16] and mitochondrial dysfunction, [17,18] which results in increased infection risk and cellular damage, thereby making survivors more vulnerable to recurrent sepsis episodes. Possible strategies to prevent recurrent infection include active surveillance of re-infections, prophylactic antibiotics, vaccination, and when possible, minimizing the use of invasive devices (*e.g.* indwelling urinary catheters, pacemakers, or intravascular lines), and avoiding drugs that suppress the immune system, such as cancer chemotherapy and direct immune suppressive drugs [19,20]. However, these strategies may not be feasible in all situations and are associated with side-effects, including the risk of antibiotic resistance, while avoiding invasive devices or immunosuppressive drugs may not be possible for those in need of these therapies. Thus, to enhance health and life span after sepsis, it is necessary to identify feasible strategies to lower the risk factors that predispose patients to recurrent sepsis episodes.

## 3 Prolonged immunosuppression

While sepsis was historically thought of as a predominantly hyper-inflammatory syndrome, recent focus has been expanded to the occurrence of an immunosuppressive phase, occurring concurrently with the hyperinflammatory phase, [21] which is marked by lymphocyte apoptosis [22] and cellular reprogramming (endotoxin tolerance) of innate immune cells [23]. Immunosuppression is evident early in sepsis, and persists after patient discharge [24]. Prolonged immunosuppression is a key component of the post-sepsis syndrome as it seems to underlie the high rate of lethal infections and sepsis recurrence [11,12]. One in five ICU sepsis survivors had positive blood cultures up to 150 days after sepsis, among which there were more opportunistic bacterial and *Candida* infections than during admission, suggesting a prolonged inability to clear infections [25]. This has important clinical consequences since, 73% of deaths in a cohort of 78 ICU sepsis survivors one year post-discharge were due to infectious complications, predominantly from pneumonia and urinary tract infections, compared to 11% in 50 non-septic ICU survivors [26]. A high frequency of lethal secondary bacterial and fungal infections in hospitalized COVID-19 patients, [27] many of whom develop sepsis, [3] suggests a similar immunosuppressive phenotype, although it is as-yet unknown how long this immunosuppression persists. Sepsis survivors have reduced pro-inflammatory interleukin-6 (IL-6) and tumor necrosis factor alpha (TNFα) secretion after stimulation of whole-blood with zymosan (a yeast surface protein), as well as a substantial decrease in anti-inflammatory IL-10 secretion in response to lipopolysaccharide (LPS) at 9-52 months after discharge, when compared to healthy controls, [24] indicating a sustained inability of immune cells to mount an effective immune response.

## 4 Mechanisms underlying sepsis-induced immune dysregulation

### 4.1 Epigenetic changes

The prolonged immunosuppressive phase may, amongst others, be explained by epigenetic mechanisms reprogramming innate and adaptive immune cells. Altered DNA methylation and histone modifications are observed in human patients and murine models post-sepsis and result in repressed expression of immune-related genes encoding TNFα, IL-1ß, IL-12, and chemokine ligand 2 (CXCL-2/MIP2-α) in macrophages and dendritic cells, [28], [29], [30] and interferon gamma (IFNγ) in CD4^+^ T-cells [31]. Murine bone marrow progenitors have repressive epigenetic modifications affecting inflammatory gene promoters four weeks after sepsis, producing macrophages resembling the impaired macrophages found in sepsis survivors [32]. This provides a potential cause as to why new innate immune cells formed after the initial septic episode appear to remain “reprogrammed”.

### 4.2 Long-term effects on immune cell numbers

Sepsis carries long-term effects on adaptive immunity. Acute sepsis leads to decreased numbers of CD4^+^ and CD8^+^ T-cells due to apoptosis, [33,34] followed by reversal to levels found in healthy individuals at six months after discharge [24]. However, despite numerical recovery of T-cells, CD4^+^ T-cells have impaired immune responses to *ex-vivo* stimulation by *Aspergillus* antigen [35] and memory CD8^+^ T-cells have decreased antigen sensitivity (as demonstrated in post-sepsis mice), [36] while stimulation of whole-blood from sepsis survivors with T-cell activator (α-CD3/28) leads to a lower IFNγ secretion as compared to healthy controls [24]. These long-term functional deficits may be due to the presence of immature neutrophils and granulocytes, called myeloid derived suppressor cells (MDSCs), which have T-cell suppressing capabilities [37]. Number of circulating MDSCs are elevated during sepsis and remain elevated up to at least four weeks after discharge [37]. Furthermore, sepsis is associated with increased number of regulatory T-cells, which persists for at least five to ten months afterwards [38]. As regulatory T-cells play an important role in dampening immune responses, their increased numbers may well contribute to persistent immunosuppression [38].

### 4.3 Immunological endotypes associated with poor long-term outcome

Recent studies have described the ability to stratify patients with sepsis into two to four different phenotypes, using (retrospective) clinical data [39] or whole-blood transcriptome data [40,41]. Stratification of septic ICU patients into four endotypes based on whole-blood transcriptome analysis identified an endotype with decreased expression of key regulators and components of the innate (*e.g.* decreased toll-like receptor expression, nuclear factor-κB and interferon signaling and antigen presentation) and adaptive (*e.g.* reduced IL-4 and T-cell signaling and overall reduction in T-/B-cell receptor signaling) immune system that was associated with the highest mortality rates, both at 28-days and one-year after discharge [40]. These genes encoding proteins involved in innate and adaptive immunity that are reduced in expression during sepsis [40] remain expressed at lower levels in sepsis survivors when compared to healthy controls [24,31]. Conversely, the endotype with the lowest mortality had increased expression of key genes involved in adaptive immune regulation (*e.g.* genes involved in T-helper cell signaling, IL-4 signaling, and B-cell development), supporting the concept that functional restoration of T-cells might reverse post-sepsis immunosuppression.

### 4.4 Therapeutic opportunities

**Fig. 1:**
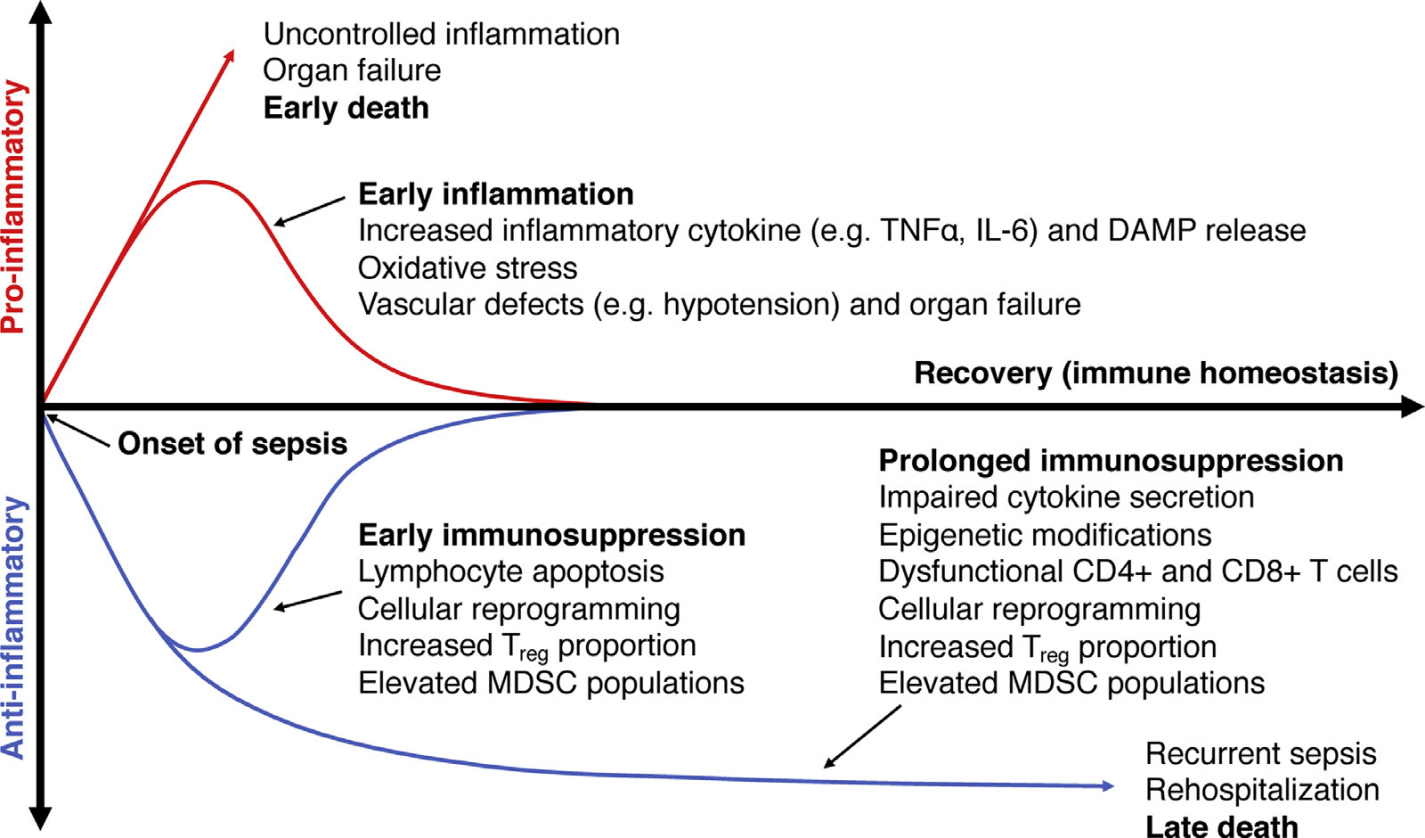
Immune dysfunction in sepsis survivors. Early in sepsis, both inflammation and immunosuppression occur concurrently. If inflammation is uncontrolled, this leads to organ failure and death. Those that avoid early death will either return to immune homeostasis, or progress to prolonged immunosuppression that continues after discharge. Prolonged immunosuppression predisposes survivors to infections, rehospitalizations, and ultimately late death. This phenomenon is marked by impaired cytokine secretion, dysfunctional T-cells, and cellular reprogramming. It is still unknown why prolonged immunosuppression occurs; however, epigenetic processes may be involved to “lock in” certain immunophenotypes. Expansion of regulatory T-cells and myeloid derived suppressor cell (MDSC) populations also occur early in sepsis and persist after sepsis, suggesting their role in maintaining this immunosuppressive phenotype. TNFα: tumor necrosis factor alpha, IL-6: interleukin-6, DAMPs: damage-associated molecular patterns, Treg: regulatory T-cell, MDSC: myeloid derived suppressor cell.

Epigenetic reprogramming of immune cells and changes in the number and function of lymphocytes appear to induce sustained immunosuppression and thereby increase susceptibility to infection in sepsis survivors (Fig. 1). Epigenetic marks can be modified *in vitro* to reprogram immune cells (*e.g.* via histone deacetylase inhibitors), [42] although such therapies have not been clinically tested. Therapies such as IL-7 or checkpoint inhibitors are currently in human trials and show potential to reverse long-term T-cell dysfunction in sepsis patients [33,43]. However, until such strategies are available, active surveillance of sepsis survivors and infectious disease control measures are the best bets to prevent recurrent episodes of sepsis.

## 5 Cognitive dysfunction

Long-term cognitive issues, with deficits in processing speed, attention span, perception, and memory, are a debilitating consequence of sepsis [7,44,45]. These deficits affect up to one in five sepsis survivors [44] and can last for up to three years [46]. Persistent cognitive deficits lead to a poorer quality of life [47] and an increased risk of rehospitalization [48]. Sepsis survivors have a reduced hippocampal volume [49] and evidence of blood brain barrier (BBB) breakdown, as detected using magnetic resonance imaging (MRI) [50]. Murine sepsis survivors have increased rates of apoptosis in hippocampal neurons, [51] increased BBB permeability, [52] and ATP depletion [53]. The occurrence of delirium in sepsis is strongly associated with long-term cognitive issues [54]. Delirium occurs in almost one in four sepsis patients [55] and approximately half of the ICU sepsis patients [56] and is associated with a high mortality rate [55]. Risk factors include acute renal failure, hyperglycemia, and electrolyte imbalances during hospitalization [57].

The association between delirium and long-term cognitive deficits might be due to permanent damage induced by cerebral inflammation and ischemia, which is part of the pathophysiology of delirium in sepsis [45,58]. Cerebral inflammation secondary to systemic inflammatory mediators (*e.g.* TNFα, IL-1β, IL-6) leads to release of damage associated molecular patterns (DAMPs, *e.g.* high-mobility group protein 1; HMGB-1) that increase BBB permeability, thereby allowing entry of cytokines into the brain, and microglial cell activation [59,60]. Neutralizing HMGB-1 one week after sepsis preserves spatial memory of mice, illustrated by better performance in a timed maze test [61]. Additionally, cerebral ischemia due to hypotension, hypoxia, and microvascular occlusion due to disseminated intravascular coagulation can cause damage, with one in three sepsis patients having (multiple) cerebral infarctions [62]. Glucose and oxygen deprivation from these infarctions leads to mitochondrial dysfunction and oxidative damage, [63] which results in neuronal apoptosis and cognitive dysfunction in septic rats [53]. Inducing mitochondrial biogenesis to increase mitochondrial mass improves cerebral ATP levels and cognition [53]. Consequently, therapies aimed at preserving cerebral mitochondrial homeostasis may prevent cognitive impairment post-sepsis.

## 6 Neuropsychiatric consequences

Severe sepsis (and other severe, acute illnesses that warrant ICU admission) can have a long-lasting effect on mental health [64,65]. Post-traumatic stress disorder (PTSD) is a common diagnosis in critical illness survivors. Nearly half of critical illness survivors suffer from PTSD at six months after discharge, which is associated with increased rates of substance abuse and sleep disturbances [65,66]. Depression and anxiety are seen in up to a third of survivors of critical illness three months after discharge [67,68]. The mental health issues of post-sepsis syndrome and “post-intensive-care unit syndrome” seem to overlap, and it is unclear whether sepsis causes any unique, lasting neuropsychiatric changes. Thus, interventions to improve mental health in ICU patients are likely applicable to sepsis patients. The exact pathology of PTSD after sepsis is unknown, although it might be triggered by severe illness and associated ICU admission [69]. Interventions to improve ICU care, such as daily sedative interruption to prevent continuous altered mental status during the ICU stay [70] or being seen by an intra-ICU clinical psychologist [71] reduces symptoms of PTSD in survivors of critical illness. Specifically for sepsis, cerebral damage may predispose to PTSD, anxiety, and depression, especially if the limbic system is affected [72]. Human sepsis survivors have signs of hypothalamic atrophy on MRIs, [49] while murine sepsis models reveal irreversible structural brain damage in the hippocampus and amygdala [50,72,73]. One intervention to manage PTSD after sepsis is keeping an ICU diary, written by healthcare workers or family during ICU stay, which is associated with a decreased incidence of PTSD (5% compared to 13% without an ICU diary) [74]. A one-year intervention involving primary care physicians and nurses trained in post-sepsis care also prevented an increase in PTSD symptoms in sepsis survivors two years after discharge [75]. The REPAIR clinical trial, which is currently in progress, will reveal whether cognitive behavioral therapy is an effective way of reducing PTSD symptoms after sepsis [64].

## 7 Cardiovascular and kidney disease

Sepsis survivors have an increased risk of fatal cardiovascular and kidney diseases, including stroke, myocardial infarction, heart failure, ventricular arrhythmia, and chronic kidney disease (CKD) [[76], [77], [78]]. The development of CKD is closely related to cardiovascular disease and may either share the same pathophysiology or be secondary to the occurrence of cardiovascular disease [77,78]. Acute kidney injury (AKI), which occurs in 30-50% of patients at the ICU and is frequently due to sepsis, [77,79] is associated with increased mortality during sepsis (67% compared to 43% in sepsis without AKI and 43% in AKI without sepsis) [80]. Similarly, patients with pre-existing CKD have a two-fold increased 90-day mortality risk when compared to septic patients without CKD [81]. Sepsis-AKI is associated with a higher risk of CKD development, [78] which also increases the risk of sepsis recurrence [81,82]. Thus, sepsis, cardiovascular, and kidney disease are closely intertwined, making it difficult to establish if patients were more prone to sepsis due to pre-existing (undiagnosed) renal/cardiac problems, or whether sepsis caused development of new problems.

The close relationship between these diseases may be explained by mitochondrial dysfunction. Sepsis causes alterations in mitochondrial architecture, damage to mitochondrial DNA, and a decrease in mitochondrial mass [18,83]. Whether mitochondrial damage is repaired after sepsis is unknown, although mice show persisting mitochondrial DNA damage four days post-sepsis [18]. Besides mitochondrial damage, sepsis is also associated with mitochondrial dysfunction (*i.e.* lowered mitochondrial membrane potential, ATP production, increased mitochondrial reactive oxygen species; ROS) [4,17,84]. Mitochondrial dysfunction seems to play a key role in the induction of sepsis-AKI, [85,86] and mitochondria-targeted antioxidants prevents AKI and lowers mortality in murine sepsis [87]. In addition, mitochondrial-targeted antioxidants decrease oxidative stress, improve mitochondrial- and organ function, and increase three day survival after sepsis in rat [87,88]. Other potential interventions include inhibition of mitochondrial ROS production to prevent mitochondrial- and cell damage, and inducing mitochondrial biogenesis to restore mitochondrial mass and oxidative metabolism [83,89]. Further implicating a key role of mitochondria during sepsis, is the impaired cardiac mitochondrial function which reduces calcium uptake leading to sarcomere destruction, contractile dysfunction and heart failure, [90,91] while renal mitochondrial dysfunction is associated with development of CKD [92,93]. Thus, mitochondrial dysfunction seems to play a key role in the pathophysiology of both sepsis, cardiovascular, and kidney diseases. Consequently, preserving mitochondrial function in sepsis may not only prevent the induction of organ injury during sepsis, but also improve long-term outcomes after sepsis.

In addition to molecular changes induced by sepsis, classic cardiovascular risk factors also increase cardiovascular and kidney disease risk among sepsis survivors. As such, obesity is associated with an increased one year mortality risk after sepsis as compared to non-obese survivors [94]. Therefore, sepsis survivors should be counseled for cardiovascular risks with attention to weight, blood pressure management, healthy lifestyle choices, and perhaps most importantly, high-density lipoprotein (HDL) management [95]. Not only do low levels of HDL and high amounts of low-density lipoprotein (LDL) increase the risk of cardiovascular events and CKD, [96,97] but low levels of HDL in (recurrent) sepsis are associated with an increased risk of organ failure, ICU admission, and mortality [96]. While the association with poor prognosis could be attributed to underlying pre-existing cardiovascular disease, sepsis itself also distorts lipid metabolism [98]. Decreased HDL levels can be used as prognostic marker for early organ failure and mortality, [98,99] which has been attributed to the ability of HDL to bind and neutralize LPS, [100] act as an immunomodulator, and preserve endothelial function [100,101]. Thus, low HDL levels increase the risk of organ failure and mortality in (recurrent) sepsis and is associated with cardiovascular and kidney disease among sepsis survivors.

Statins and modulation of HDL levels might reduce the risk of cardiovascular events among sepsis survivors. However, cholesterol management remains controversial since low levels of LDL are also associated with an increased sepsis risk [102]. One reason cholesterol management may work is that persistent, low-grade systemic inflammation in sepsis, that can occur simultaneously with immunosuppression, [16,103] may destabilize atherosclerotic plaques which could lead to plaque rupture and cause a stroke or myocardial infarction [104,105]. Pre-treatment of mice with statins before sepsis[106], [107], [108], or after sepsis [109], improves survival, possibly due to plaque stabilization combined with decreased ROS production and immunomodulatory effects [106,110]. Additionally, treatment of mice with statins after sepsis lowers neuroinflammation, endothelial dysfunction, and cognitive decline [111] and statins use in patients with atherosclerosis is associated with a reduced sepsis risk [110]. Experimental modulation of HDL levels by administration of ApoA1-mimetic peptides or reconstituted HDL in animal models of sepsis decreases inflammation, organ damage, and mortality [101,112]. Since a gain-of-function mutation in cholesteryl ester transfer protein (CETP) is associated with lower HDL levels and higher mortality in sepsis, [113] increasing HDL levels using CETP inhibitors seems to be another promising strategy [113]. Whether restoring HDL levels will also reduce cardiovascular and kidney disease among sepsis survivors is as-yet unknown. Overall, sepsis survivors suffer from a high risk of cardiovascular and kidney disease, although it is unclear if the underlying pathophysiology is the same as non-sepsis-associated development of cardiovascular and kidney disease. If not, classic cardiovascular risk management strategies may be insufficient to prevent cardiovascular problems after sepsis and the focus of new therapies should move towards targeting underlying mechanisms, including mitochondrial dysfunction.

## 8 Overall functioning and quality of life

Sepsis survivors continue to have a reduced health-related quality of life (QoL) for at least five years after discharge, particularly in the physical domain, when compared to age-matched controls [114]. Similarly, almost half of acute COVID-19 survivors reported decreased QoL 60 days after first onset of symptoms compared to before developing COVID-19, mainly due to fatigue and joint pain [115]. Decreased physical function may be due to loss of muscle mass during sepsis, though it is incompletely understood why muscle regeneration is impaired after sepsis [116]. Mitochondrial dysfunction, as seen in in muscle stem cells in septic mice, may well underlie impaired muscle regeneration in sepsis survivors [18]. Consequently, poor physical functioning leads to inability to work in more than half of previously-employed sepsis survivors [116]. A poor QoL six months after sepsis is predictive of a worsening QoL one year after sepsis; [117] therefore, it is key to identify patients with early decreases in QoL who may need closer follow-up and personalized strategies to improve QoL.

Physiotherapy can improve physical QoL after sepsis. Initiation of physical rehabilitation within three months after discharge not only improved physical strength, but also reduced ten-year mortality in sepsis survivors, as compared to sepsis survivors who did not receive physiotherapy [118]. Furthermore, early mobility interventions improve physical function at discharge, as compared to patients who received only primary care during sepsis [119]. The benefits of physiotherapy are likely mediated by improved mitochondrial function and reduced inflammation, which then improves both physical and cognitive health [120,121]. Based on the molecular mechanisms underlying the reduced physical function after sepsis in mice, [18] mesenchymal stem cell therapy seems to be a promising adjuvant future therapy to improve muscle strength and overcome impaired muscle regeneration via restoration of mitochondrial function in muscle cells.

## 9 Current and future therapeutic opportunities to optimize long-term outcome after sepsis

**Fig. 2:**
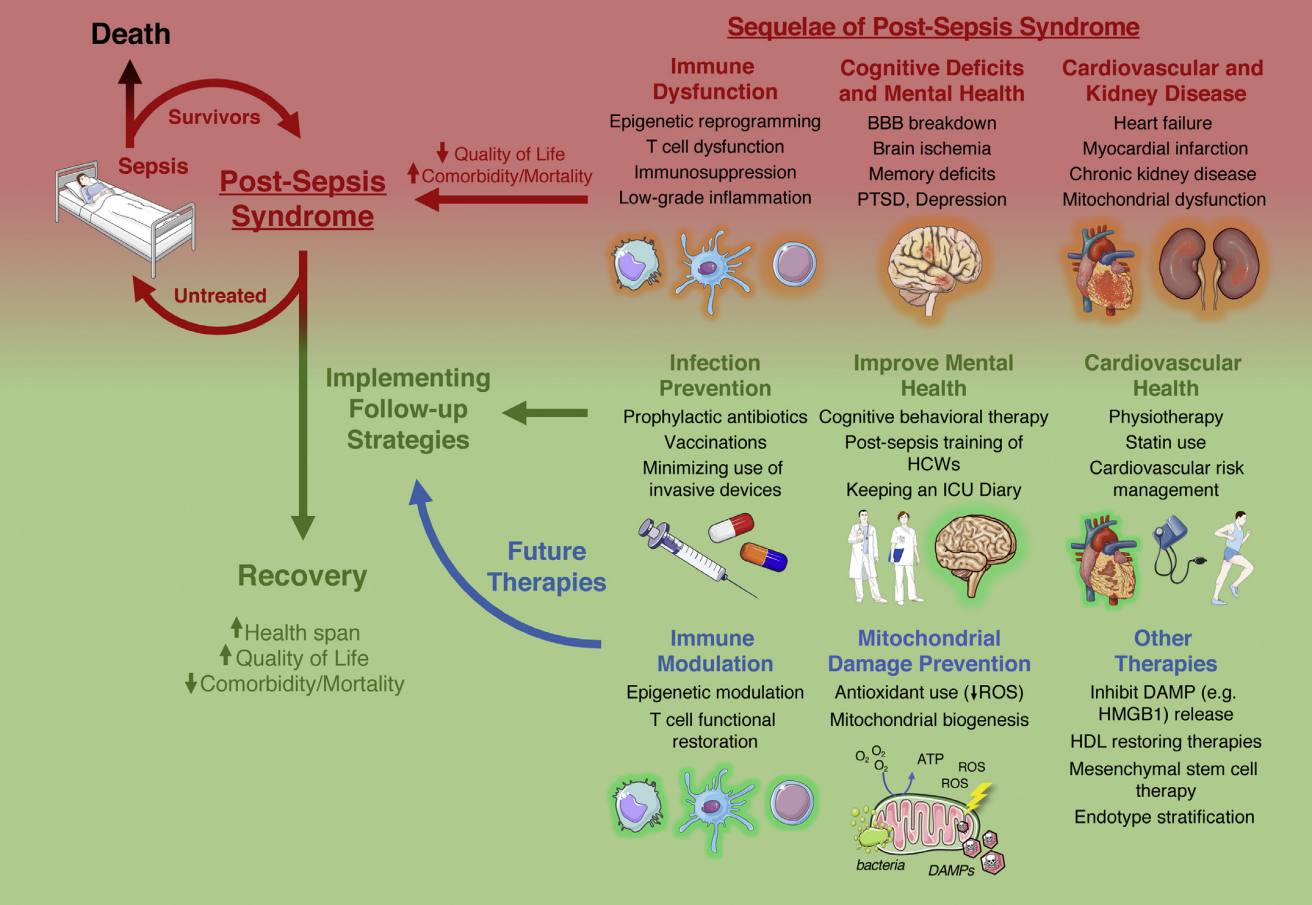
Current and future strategies to combat post-sepsis syndrome**.** Many sepsis survivors suffer from at least one aspect from post-sepsis syndrome, which is characterized by immune dysfunction, cognitive deficits, mental health problems, and cardiovascular/kidney disease, causing decreased quality of life and mortality. Left untreated, post-sepsis syndrome will lead to rehospitalization and recurrent sepsis, placing the patient in a lethal feedback loop. Follow up strategies include infection control, improving mental health, cardiovascular risk management, statins, and physiotherapy. Future therapies include reversing immune dysfunction, prevention of mitochondrial damage, inhibiting DAMP release, HDL restoring therapies, mesenchymal stem cell therapy and risk stratification based on endotype. (BBB: blood brain barrier, PTSD: post-traumatic stress disorder, HCW: health care worker, ICU: intensive care unit, DAMP: damage-associated molecular pattern, ROS: reactive oxygen species). Fig. 2 was created with images from Servier Medical Art (http://smart.servier.com), licensed under a Creative Common Attribution 3.0 Generic License.

Post-sepsis syndrome consists of immunological, cardiovascular, and cognitive deficits that persist long after hospital discharge, resulting in more frequent rehospitalizations due to recurrent sepsis, decreased QoL, and increased comorbidity and mortality (Fig. 2). A sepsis follow-up clinic seems to be a useful strategy to allow doctors and scientists to provide post-sepsis care while collecting relevant data from sepsis survivors, performing clinical trials to determine optimal post-sepsis rehabilitation strategies, and expanding insights into the mechanisms that underlie the long-term consequences of this syndrome. We propose that endotype stratification during sepsis, based on clinical and/or molecular data, can identify patients at increased risk for the development of post-sepsis syndrome; [40] this strategy will also expand fundamental knowledge about the pathophysiology of post-sepsis syndrome with relevance to the development of novel therapies. In addition, mitochondrial dysfunction is linked to the development and progression of chronic diseases particularly after sepsis, including cardiovascular, neurodegenerative, and kidney disease [18,63,85]. Consequently, preventing mitochondrial damage during sepsis or restoring mitochondrial function could counteract the long-term effects of sepsis on health and life span. Table 1 summarizes the clinically relevant long-term consequences after sepsis and novel treatments that might resolve or prevent these sequelae.

**Table 1:** Post-sepsis syndrome sequelae, their proposed pathophysiology, and current or future strategies that target these pathophysiological mechanisms.

Post-Sepsis Syndrome Sequelae	Proposed Pathophysiology	Current Strategies	Future Strategies
Persistent immune dysfunction,[24] leading to recurrent infections and sepsis[14,26]	Epigenetic reprogramming[28,[30], [31], [32]] T cell dysfunction[24,35,36] Cellular reprogramming[24] Increased MDSCs[37] Increased regulatory T cells[38]	Promoting infection control practices Prophylactic antibiotics Vaccinations Minimizing use of invasive devices	Reversal of epigenetic reprogramming[42] IL-7 therapy[33] Checkpoint inhibitors[43] Risk stratification based on endotype[39], [40], [41]
Cognitive impairment	Cerebral inflammation[60,61] Cerebral ischemia,[62] leading to mitochondrial dysfunction[50]	Sending patients to long-term care homes	Targeting DAMPs (*e.g.* HMGB-1) to prevent cerebral damage[61] Reversing mitochondrial dysfunction (*e.g.* mitochondrial biogenesis)[53]
Post-traumatic stress disorder	Traumatic experiences in the ICU[69] Cerebral damage, especially in the limbic system[72]	Avoiding continuous sedative use in ICU[70] Being seen by an ICU psychologist[71] Care by healthcare workers trained in post-sepsis care[75] Maintaining an “ICU diary”[74] Cognitive behavioural therapy[64]	Preventing or reversing cerebral damage[53,61]
Cardiovascular disease	Mitochondrial dysfunction Cardiovascular risk factors (e.g. high BMI, blood pressure, cholesterol levels)[4,17,18,83]	Weight, blood pressure, and cholesterol management[94,95] Physical activity[121] Statin use[108,110]	Antioxidants[87] Reversing mitochondrial dysfunction (inhibition of mitochondrial ROS production, increasing mitochondrial biogenesis)[83,89] HDL increasing agents[100,101,113]
Decreased quality of life	Impaired muscle regeneration due to mitochondrial dysfunction[18]	Physiotherapy and physical rehabilitation[118,119]	Mesenchymal stem cell therapy[18]

## 10 Conclusion

Overall, the pathophysiology of sepsis and post-sepsis syndrome remains poorly understood mainly due to its heterogeneous nature, thereby making it hard to treat. Injury occurring during sepsis is likely only partially repaired, leaving sepsis survivors with post-sepsis syndrome. Therefore, we should realize that sepsis is more than an intermittent acute disease. Long-term effects of the post-sepsis syndrome consist of persistent immune, cognitive, neuropsychiatric, and cardiovascular dysfunctions, resulting in frequent rehospitalization, increased mortality, and decreased quality of life compared to survivors of other acute medical conditions. Understanding the pathophysiology of these aspects of post-sepsis syndrome has led to the development of the mechanism-guided therapies listed in this review; however, few clinical trials have been done to test these interventions, perhaps due to the difficulty of finding, enrolling, and following up with sepsis survivors, problems which a post-sepsis clinic may alleviate. Immune endotypes and mitochondrial dysfunction seem to be of substantial importance in defining patient outcomes and improving those features using future therapies might ultimately improve the health and life span of sepsis survivors.

## Outstanding questions

1. What is the efficacy of using post-sepsis clinics to alleviate symptoms of post-sepsis syndrome?
2. Which specialties should be involved in the multidisciplinary post-sepsis team?
3. What role does mitochondrial dysfunction play in multiple aspects of post-sepsis syndrome?
4. What is the best way to stratify sepsis survivors to individualize follow-up care? Could endotype stratification be a possibility?
5. With emerging evidence of COVID-19 survivors with persistent symptoms similar to post-sepsis syndrome, are these two phenomena related, and if so, can therapeutics outlined in this review help COVID-19 survivors?

## Search strategy and selection criteria

Articles for this review were identified using PubMed, Google Scholar, and references from relevant articles using the search terms: ‘Sepsis’ OR ‘Post-sepsis syndrome’, AND ‘Rehospitalization’ OR ‘Long-term outcome’ OR ‘Immune system’ OR ‘Quality of life’ OR ‘Cognitive dysfunction’ OR ‘Cardiovascular system’ OR ‘Chronic kidney disease OR ‘Psychiatric disorder’. Only the most impactful papers were considered.

## Declaration of Competing Interests

REWH reports grants from Canadian Institutes of Health Research covering sepsis research, during the writing of this review. REWH is a major shareholder of the virtual private company Sepset Biotherapeutics Inc, Vancouver, British Columbia, that is developing diagnostics for early sepsis, and has a patent (“Diagnostic for Sepsis”, US20200032321A1) licensed to Sepset Biotherapeutics Inc. Dr. Bouma reports grants from Dutch Kidney Foundation, during the conduct of the study. The other authors declare no conflict of interests.
